# Prevalences of pathogenic and non-pathogenic *Vibrio parahaemolyticus* in mollusks from the Spanish Mediterranean Coast

**DOI:** 10.3389/fmicb.2015.00736

**Published:** 2015-07-21

**Authors:** Carmen Lopez-Joven, Ignacio de Blas, M. Dolores Furones, Ana Roque

**Affiliations:** ^1^IRTA-SCRSant Carles de la Ràpita, Spain; ^2^Laboratory of Fish Diseases, Veterinary Faculty, Universidad de ZaragozaZaragoza, Spain

**Keywords:** prevalence, human-pathogenic variants, *tdh*, *trh*, *Vibrio parahaemolyticus*, bivalves, salinity, water temperature

## Abstract

*Vibrio parahaemolyticus* is a well-recognized pathogen of humans. To better understand the ecology of the human-pathogenic variants of this bacterium in the environment, a study on the prevalence in bivalves of pathogenic variants (*tlh*+ and *tdh*+ and/or *trh*+) versus a non-pathogenic one (only *tlh*+ as species marker for *V. parahaemolyticus*), was performed in two bays in Catalonia, Spain. Environmental factors that might affect dynamics of both variants of *V. parahaemolyticus* were taken into account. The results showed that the global prevalence of total *V. parahaemolyticus* found in both bays was 14.2% (207/1459). It was, however, significantly dependent on sampling point, campaign (year) and bivalve species. Pathogenic variants of *V. parahaemolyticus* (*tdh*+ and/or *trh*+) were detected in 3.8% of the samples (56/1459), meaning that the proportion of bivalves who contained *tlh* gene were contaminated by pathogenic *V. parahaemolyticus* strains is 27.1% (56/207). Moreover, the presence of pathogenic *V. parahaemolyticus* (*trh*+) was significantly correlated with water salinity, thus the probability of finding pathogenic *V. parahaemolyticus* decreased 1.45 times with every salinity unit (ppt) increased. Additionally, data showed that *V. parahaemolyticus* could establish close associations with *Ruditapes* spp. (*P-value* < 0.001), which could enhance the transmission of illness to human by pathogenic variants, when clams were eaten raw or slightly cooked. This study provides information on the abundance, ecology and characteristics of total and human-pathogenic *V. parahaemolyticus* variants associated with bivalves cultured in the Spanish Mediterranean Coast.

## Introduction

*Vibrio parahaemolyticus* is a bacterium commonly present in marine and estuarine water worldwide (Kaneko and Colwell, 1975).

The virulence of *V. parahaemolyticus*, among other virulence attributes, depends on the presence of a thermostable direct hemolysin (TDH, *tdh* gene) and/or the thermostable direct hemolysin related (TRH, *trh* gene; Takeda, 1982; Shirai et al., 1990; Honda and Iida, 1993; Oliver and Kaper, 2007; Ceccarelli et al., 2013; Raghunath, 2015). Despite that the bacterium can be found naturally in seafood and taking into account that bivalves are filter feeders and accumulate environmental bacteria in their gills and digestive glands becoming potential vectors for many pathogens (Potasman et al., 2002), pathogenic isolates capable of inducing gastroenteritis in humans are rare in environmental samples (2 to 3%) or even undetectable (Nair et al., 2007; Canizalez-Roman et al., 2011; Velazquez-Roman et al., 2012; Haley et al., 2014). It should be noted that, in recent studies using a new set of primers have shown that higher frequencies of the *tdh* and *trh* genes can be detected in environmental *V. parahaemolyticus* strains than primers described previously (Gutierrez West et al., 2013). Anyway, despite that only, few cases of gastroenteritis by *V. parahaemolyticus* have been reported so far in Europe, there is a growing concern on that non-cholera vibrios could represent an important and increasing clinical threat within the European context (Baker-Austin et al., 2010). Furthermore, the scenario could worsen by climate global change which plays an important role in the dissemination of pathogenic microorganism in the marine environment (Baker-Austin et al., 2013; Huehn et al., 2014; Letchumanan et al., 2014).

This study examines the spatial distribution and temporal changes of the total and pathogenic *V. parahaemolyticus* in the delta of Ebro River, Catalonia, Spain. Catalonia which is the second-most important region of Spain in terms of bivalve production, being Spain the second largest producer in the world and one of the largest consumers of bivalves (APROMAR, 2011; Eurostat, 2012). Farming of bivalves in Catalonia is concentrated in the two bays (Alfacs and Fangar) in the delta of the Ebro River. The average and ranges of water temperature and salinity, along with the moderately alkaline pH of the two bays provided the conditions to support growth of vibrios (Montilla et al., 1994). The risk of potentially pathogenic *Vibrio* spp. in products placed on the market is not addressed by the existing legislative framework related to food safety in the European Union. However, it is recognized, the need for a better knowledge of the prevalence of diarrheal vibrios in seafood products (European Commission, 2001).

This study is one of the few that has focused on the examination of large numbers of oysters, mussels, and clams with the objective to investigate the prevalence, spatial distribution and temporal change of total and pathogenic *V. parahaemolyticus* associated to different aquaculture bivalves in the Ebro delta and its relationship with environmental parameters from the surrounding waters.

## Materials and Methods

### Sampling Sites and Collection of Samples

**FIGURE 1:**
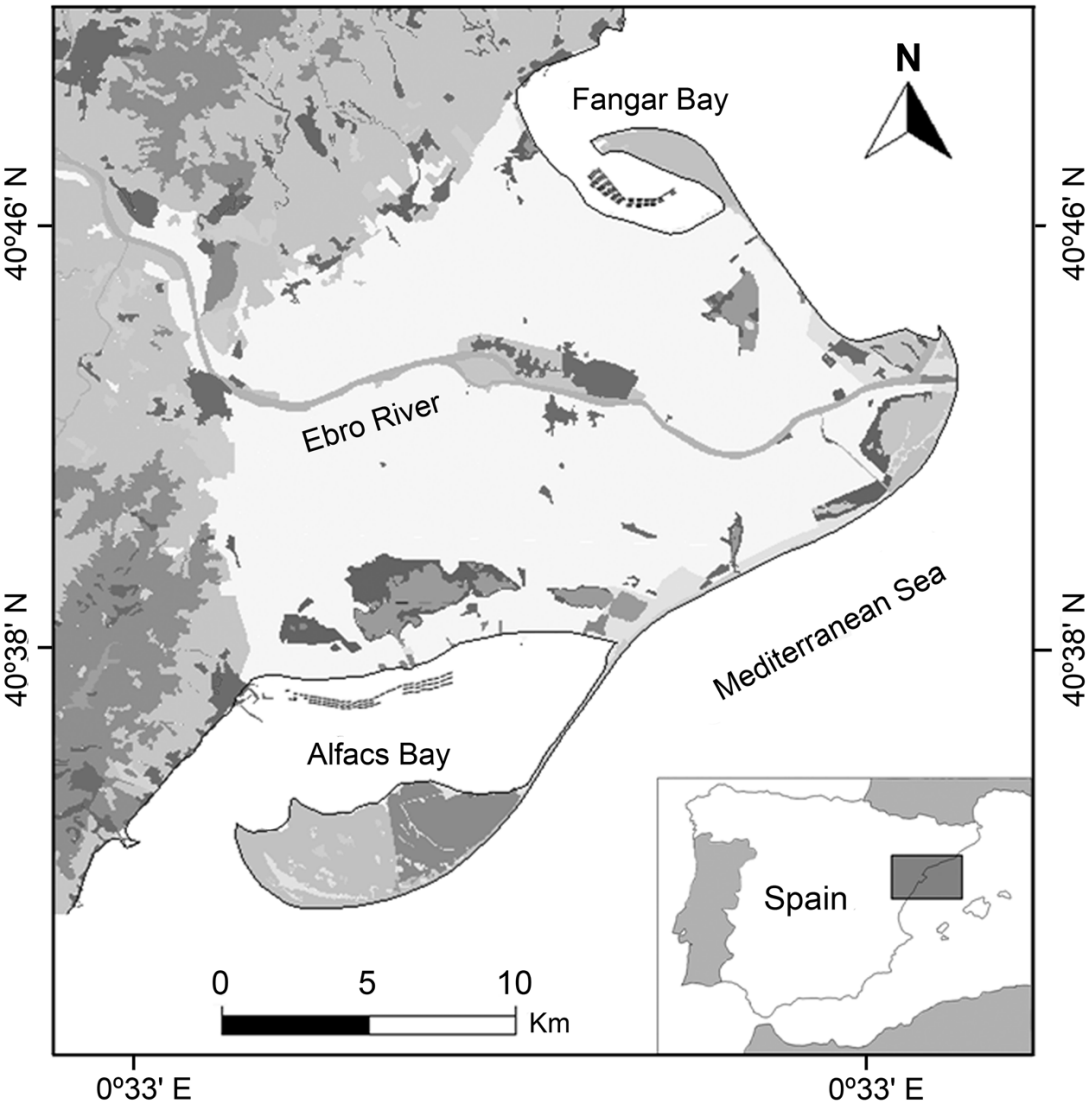
**Area of study showing: location of the sampling sites in the Ebro Delta, Spain**.

Four surveillance campaigns for pathogenic *V. parahaemolyticus* detection in commercial bivalves from the Ebro delta bays (**Figure 1**) were performed from 2006 to 2010 (see Roque et al., 2009 for details).

The sample size was established to achieve a high confidence level (CL), between 95 and 99%, considering that some shellfish are eaten raw or lightly cooked. Thus, the minimum expected prevalence for mussels and oysters was 5 and 10% for clams, where sample availability was limited (**Table 1**). Unfortunately, was not possible to carry out the survey in 2007.

**Table 1:** Surveillance scheme in order to calculate sample size for different mollusk species in each bay.

	2006			2008			2009			2010		
Species	*n*	CL	mEP	*n*	CL	mEP	*n*	CL	mEP	*n*	CL	mEP
*Crassostrea gigas*	90	99%	5%	90	99%	5%	90	99%	5%	60	95%	5%
*Mytilus galloprovincialis*	60	95%	5%	90	99%	5%	90	99%	5%	60	95%	5%
*Ruditapes* spp.	30	95%	10%	30	95%	10%	30	95%	10%	30	95%	10%

*n, calculated sample size; CL, confidence level; mEP, minimum expected prevalence.*

Oysters and mussels samples were collected in each bay, from at least three ropes, at three production units (*fixed* platforms with hanging ropes), while clams were sampled in three different points in one culture area (clam field) in each bay. Mussel and oysters were sampled from six sites of the culture area, three on each bay, at the beginning (N 40°37′112″E0°37′092″-Alfacs; N40°46′723″E0°43′943″-Fangar), middle (N 40°37′125″E0°38′570″-Alfacs; N40°46′666″E0°45′855″-Fangar), and end (N 40°37′309″E0°39′934″-Alfacs; N40°46′338″E0°44′941″-Fangar) of the culture polygons. Clams were sampled from only one site per bay, in the Alfacs bay from a natural bed of *Ruditapes decussatus* (N40°37′44″E0°38′0″) and in the Fangar bay from an aquaculture bed of *Ruditapes philippinarum* (N40°47′3″E0°43′8″; **Figure 1**).

Some deviations from the sample size of the surveillance scheme (**Table 1**) were originated due to lack of adult specimens, and therefore they had to be discarded.

A total of 1459 species of bivalve mollusks were sampled: 709 and 750 bivalves from Alfacs and Fangar Bays, respectively. From the 709 individuals collected in Alfacs Bay, 283 were oysters (*Crassostrea gigas*), 306 were mussels (*Mytilus galloprovincialis*), and 120 clams (*Ruditapes* spp.). And, from the 750 individuals collected from Fangar Bay, 330 oysters, 300 mussels, and 120 clams were processed. So, similar proportional number for each species was taken from the both bays and for each year of study as shown in **Table 2**. On the whole, oyster’ samples were 180, 178, 150, and 105 in 2006, 2008, 2009, and 2010, respectively. Mussels samples were 127, 179, 180, and 120 in 2006, 2008, 2009, and 2010, respectively, and clams samples were 60 for each year.

**Table 2:** Prevalence of total *V. parahaemolyticus*, stratified by years, by bays, and by species of bivalve.

	2006				2008				2009				2010			
	Alfacs		Fangar		Alfacs		Fangar		Alfacs		Fangar		Alfacs		Fangar	
	*n*	*Vp* %	*n*	*Vp* %	*n*	*Vp* %	*n*	*Vp* %	*n*	*Vp* %	*n*	%	*n*	%	*n*	%
*Crassostrea gigas*	90	12.2%	90	25.6%	88	10.2%	90	21.1%	60	11.7%	90	11.1%	45	4.4%	60	11.7%
*Mytilus galloprovincialis*	67	4.5%	60	6.7%	89	11.2%	90	37.8%	90	8.9%	90	2.2%	60	5.0%	60	15.0%
*Ruditapes decussatus*	30	56.7%	–	–	30	30.0%	–	–	30	3.3%	–	–	30	6.7%	–	–
*Ruditapes philippinarum*	–	–	30	0.0%	–	–	30	40.0%	–	–	30	16.7%	–	–	30	0.0%
**Total**	**187**	**16.6%**	**180**	**15.0%**	**207**	**13.5%**	**210**	**31.0%**	**180**	**8.9%**	**210**	**8.1%**	**135**	**5.2%**	**150**	**10.7%**
*P*-value^1^	<0.001		<0.001		0.017		0.027		0.424		0.016		0.914		0.089	
*P*-value^2^	<0.001				0.010				0.070				0.125			

*^1^*P*-value: prevalences of total *V. parahaemolyticus* (*tlh+*) stratified by years and by bays; ^2^*P*-value: Prevalences of total *V. parahaemolyticus* (*tlh+*) stratified by years.*

All bivalves were collected during the warmest season (July and August) each sampling year. Samples were transported in cool conditions to the laboratory. Transport lasted less than 1 h in all occasions.

Temperature and salinity data were recorded using a CTD datalogger (Sea-Bird, USA) located in the center of each bay at a depth of 2 m and recording data every 60 min. The center of each bay was selected since it coincides with the middle point of the area covered by the culture rafts.

### Microbiological Analysis

Microbiological analysis was carried out as described by Roque et al. (2009). Briefly, all bivalves were individually homogenized and processed and 1 ml of the homogenate was inoculated into 9 ml of alkaline peptone water (Scharlau, Spain). Following 6 h incubation at 37°C, one loopful of the contents of each tube of alkaline peptone water was streaked onto CHROMagar vibrio plates (CHROMagar, France) and incubated for 18 h at 37°C. Mauve-purple colonies were purified, and each purified isolate was cryopreserved at -80°C for further analysis.

### Molecular Tests

Total DNA was extracted from each purified isolate using the Wizard genomic DNA purification kit (Promega), following the instructions of the manufacturer. DNA concentration was verified by spectrophotometry and the concentration of each DNA was adjusted to 50 ng μl^-1^. The PCR analysis was then performed to identify which isolates were positive for *tlh* gene (species marker for *V. parahaemolyticus*). The further detection of the *tdh* or *trh* genes was carried out on all positive *tlh* strains. All PCR analyses were carried out using the primers described by Bej et al. (1999) with the following amplification conditions on the thermocycler (Eppendorf Mastercycler Personal, Hamburg, Germany): an initial denaturation at 95°C for 8 min, followed by 40 cycles of a 1 min denaturation at 94°C, annealing at 55°C for 1 min, elongation at 72°C for 1 min, and a final extension of 10 min at 72°C. Positive and negative controls were included in all reaction mixtures: two positive controls, *tlh* and *tdh* CAIM 1400 and *trh* CAIM 1772 [Collection of Aquatic Important Microorganisms (http://www.ciad.mx/caim/CAIM.html)], and negative control DNA-free molecular grade water (Sigma-Aldrich, Spain). Expected amplicons were visualized in 2% agarose gels stained with ethidium bromide.

### Statistical Analysis

The influence of different factors such as species of mollusk, location (Alfacs or Fangar) and year of study on prevalences (proportion of mollusks contaminated by *V. parahaemolyticus*) was assessed by Pearson’s Chi-Square test.

A logistic regression analysis was performed using *V. parahaemolyticus* prevalence as dependent variable and year, location, mollusk species, salinity, and temperature as independent variables. Forward stepwise method was applied, and significance of the model was evaluated with omnibus test. Using coefficients of the model (Bx) Odds Ratio (OR = e^Bx^) were calculated to identify significant risk factors.

Desired alpha error was established at 0.05. Statistical analysis was performed using SPSS 19.0 software (Chicago, IL, USA).

All data collected represents data points and not a continuous variable.

## Results

During four summer surveillance campaigns (years 2006, 2008, 2009, and 2010), a total of 1459 bivalve mollusks (*C. gigas, M. galloprovincialis, R. decussatus*, and *R. philippinarum*) at commercial size, were collected and processed in the two shellfish production areas of Alfacs and Fangar Bays of the delta of Ebro river to assess the prevalences of *V. parahaemolyticus*.

### Global Prevalence of *V. parahaemolyticus*

**FIGURE 2:**
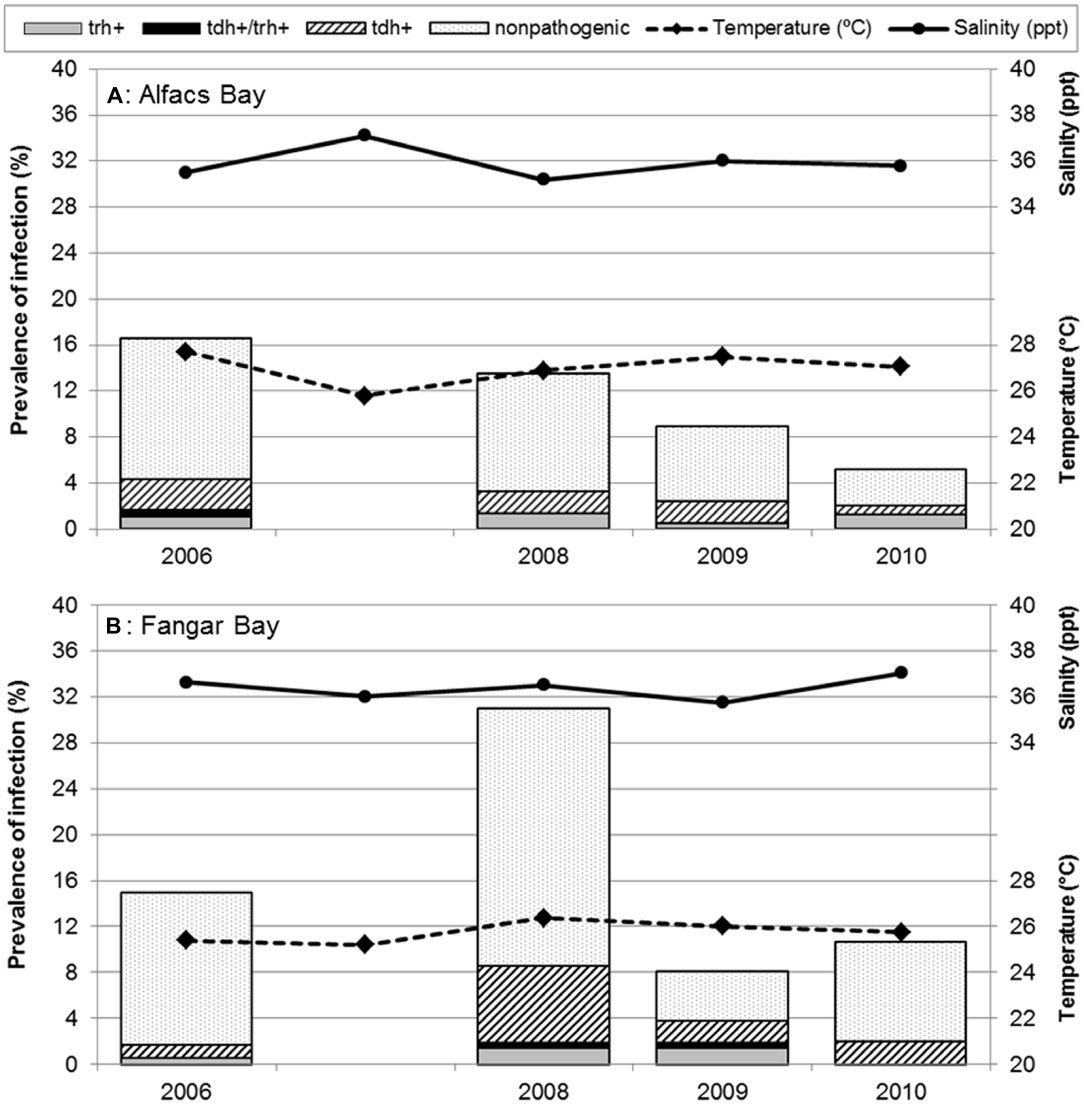
**Prevalence of pathogenic and non-pathogenic *Vibrio parahaemolyticus* in Mediterranean mollusks: stratified results in **(A)** Alfacs Bay and **(B)** Fangar Bay.** Each bar represents the arithmetic mean of four species of bivalves averaged in sampling months. Solid line represents salinity (ppt); dashed line, water temperature (°C) during each campaign.

Different trends in non-pathogenic and pathogenic *V. parahaemolyticus* prevalences and their relationship with water temperature (°C) and salinity (ppt) from both sampling sites, are shown in **Figure 2**. Overall, in the two bays, *V. parahaemolyticus* was detected in 207 (14.2%) of the 1459 samples identified by targeting thermolabile hemolysin encoded by *tlh* gene. When statistical analysis was performed to compare prevalence of *V. parahaemolyticus* carrying the *tlh* gene among sampling bays, they were found to be significantly different (*P* = 0.005), being Alfacs’ prevalence (11.6%, 82/709) lower than Fangar’s (16.7%, 125/750). When the analysis was stratified also by year, significant differences were observed only in 2008 (*P* < 0.001), where the proportion of bivalves mollusks from Fangar with *V. parahaemolyticus* (31.0%) was higher than in Alfacs (13.5%). No differences due to the sampling site were detected in 2006 (*P* = 0.679), in 2009 (*P* = 0.779), or in 2010 (*P* = 0.090) though prevalence of total *V. parahaemolyticus* in Fangar (10.7%) were double than Alfacs (5.2%). When prevalences of total *V. parahaemolyticus* were analyzed in each bay over the sampling period, it was observed that the prevalence in Alfacs Bay decreased significantly (*P* = 0.007) over the studied period: 2006 (16.6%), 2008 (13.5%), 2009 (8.9%), 2010 (5.2%); while, the prevalence of total *V. parahaemolyticus* in Fangar bay was significantly different among years (*P* < 0.001), but fluctuating over time: 2006 (15.0%), 2008 (31.0%), 2009 (8.1%), and 2010 (10.7%; **Figure 2**).

Prevalence of pathogenic *V. parahaemolyticus* were studied considering the different trends of their virulence factors in relation with water temperature and salinity at the sampling sites as shown in **Figure 2**. The global prevalence of total mollusks contaminated by pathogenic *V. parahaemolyticus* (with virulence genes *tdh, trh*, or both) was estimated to be 3.8% (56/1459).

When stratified analysis of prevalence of pathogenic *V. parahaemolyticus* by bays was performed, no significant differences (*P* = 0.251) were observed between Alfacs (3.2%) and Fangar (4.4%). In both cases, proportion of bivalves contaminated by *V. parahaemolyticus* that carried pathogenic strains were similar, and the overall prevalence was 27.1%, with the following distribution: *tdh* (17.9%), *tdh/trh* (1.4%) and *trh* (7.7%), meaning that from the 212 mollusks containing *V. parahaemolyticus* (*tlh*+), a total of 57 also presented a virulence factor (*tdh, trh*, or both).

Globally, significant differences were observed (*P* = 0.053) when the presence of virulence factors was investigated over time in both bays together. Results showed higher proportion of the bivalves contained pathogenic *V. parahaemolyticus* in 2008 (6.0%, 25/417) than in other years, mainly, due to the contribution of Fangar Bay. Also, when taking into account the stratified analysis by year and by bays for the virulence factors, no significant differences were observed in Alfacs (*P* = 0.747), in contrast to Fangar. Here, significant differences were recorded (*P* = 0.004), with the highest prevalence of pathogenic *V. parahaemolyticus* being 8.6% in 2008, of which 6.7% were *tdh*-positive, 1.4% were *trh*-positive, and 0.5% were *tdh/trh*-positive (**Figure 2**).

Regarding *V. parahaemolyticus* strains harboring the *trh* gene, the highest prevalence observed was 1.4% in both bays in the whole studied period (this value was recovered in 2008 in Alfacs, and in 2008 y 2009 in Fangar).

Prevalences of total *V. parahaemolyticus*, stratified by years, by bays, and by species of bivalves are shown in **Table 2**. Prevalences among species of bivalves in Alfacs Bay, were different in 2006 (*P* < 0.001), and in 2008 (*P* = 0.017), where prevalence in *R. decussatus* (56.7 and 30.0%, respectively), was higher than in *C. gigas* or *M. galloprovincialis*, but this was not the case in 2009 (*P* = 0.424) and 2010 (*P* = 0.914).

In Fangar Bay, significant difference were found in 2006 (*P* < 0.001), where higher prevalence was found in *C. gigas* (25.6%). In 2008 (*P* = 0.027), prevalences were higher in *R. philippinarum* (40.0%) and in *M. galloprovincialis* (37.8%), and in 2009 (*P* = 0.016), they were higher in *R. philippinarum* (16.7%). No significant differences (*P* = 0.089) were found in prevalences of total *V. parahaemolyticus* in mollusks in 2010.

*Vibrio parahaemolyticus* was not present in all sample batches in Fangar bay, e.g., *V. parahaemolyticus* was not detected in *R. philippinarum* neither in 2006 nor in 2010.

When prevalences of pathogenic *V. parahaemolyticus* by species of bivalves were analyzed for either bay, no significant differences were found (Alfacs *P* = 0.065; Fangar *P* = 0.822) during the four campaigns.

When statistical analysis was performed to study if global prevalence of tot *V. parahaemolyticus* among species of bivalve were significantly different (without stratify by bay or year), results (see **Table 3A**) indicated that significant differences were found among species (*P* = 0.007). The total prevalence by mollusk species were as follows: 14.4% (88/613) in oysters (*C. gigas*), 12% (73/606) in mussels (*M. galloprovincialis*), 24.2% (29/120) in *R. decussatus*, and 14.2% (17/120) in *R. philippinarum*. In this context of stratification by species, prevalences of total *V. parahaemolyticus* were significant different within mussels (*P* < 0.001), and within both species of clams (*P* < 0.001), whereas results for oysters did not show any significant differences (*P* = 0.064).

**Table 3:** **(A)** Prevalence of total *V. parahaemolyticus* (*tlh*^+^); and **(B)** Prevalence and relative frequency of pathogenic *V. parahaemolyticus* (*tdh*^+^ and/or *trh*^+^), both of them stratified by species of bivalves mollusks.

	Sampled mollusks	(A) Prevalence total *Vp*			(B) Prevalence pathogenic *Vp*			% Pathogenic *Vp*
	*n*	*n*	%	*P-value*^2^	*n*	%	*P-value*^3^	
*Crassostrea gigas*	613	88	14.4%	0.064	26	4.2%	0.081	29.5%
*Mytilus galloprovincialis*	606	73	12.0%	<0.001	19	3.1%	0.052	26.0%
*Ruditapes decussatus*	120	29	24.2%	<0.001	7	5.8%	0.134	24.1%
*Ruditapes philippinarum*	120	17	14.2%	<0.001	4	3.3%	0.009	23.5%
**Total**	**1459**	**207**	**14.2%**	**<0.001**	**56**	**3.8%**	**0.053**	**27.1%**
*P-value*^1^		0.007			0.506		0.908

*^1^*P*-value: prevalence of total *V. parahaemolyticus*, stratified by years; ^2^*P*-value: prevalence of total *V. parahaemolyticus (tlh+)*, stratified by species of mollusks; ^3^*P*-value: prevalence of pathogenic *V. parahaemolyticus* (*tdh+* and /or *trh+*), stratified by species of mollusks.*

When global prevalence and relative frequency of pathogenic *V. parahaemolyticus* (*tdh* and/or *trh*+) stratified only by species on bivalve were analyzed (**Table 3B**), the results showed also no significant differences (*P* = 0.506); being the prevalences as follows: 4.2% in oysters (26/613), 3.1% in mussels (19/606), 5.8% in *R. decussatus* (7/120), and 3.3% in *R. philippinarum* (4/120). The same happened when only contaminated bivalves with total *V. parahaemolyticus* (207/1459 total samples) were taken into account, meaning 56/207 bivalves mollusks (*P* = 0.908).

### Multivariate Analysis of Influence of Different Factors on the Presence of Total *V. parahaemolyticus*

The relationship between the prevalence of total *V. parahaemolyticus* with species of bivalves, shell length (mm), year of harvest, sampling bay, water temperature, and salinity was analyzed using a logistic regression with Forward Stepwise method.

Two multivariate analysis were used, one including sampling bay and the other without it. A significant logistic regression model that included bay to explain the presence/absence (P/A) of total *V. parahaemolyticus* in bivalves was generated (*P* < 0.001; see **Table 4** for details), and it established that the presence of *V. parahaemolyticus* depends on location of the samples (Alfacs or Fangar), species of mollusks, and year of harvest. Effect of sampling bay was significant (*P* < 0.001) and the risk of finding *V. parahaemolyticus* in mollusks from Fangar Bay was 2.21 times higher than from Alfacs Bay. Moreover, results showed that year of sampling were significant using as reference year 2006. Thus, the risk (expressed as Odds Ratio) to find total *V. parahaemolyticus* varied with time. Results obtained in 2008 respect to 2006 indicated that the risk to find total *V. parahaemolyticus* in bivalves was 1.57 times higher in 2008 than 2006 (*P* = 0.017), and these results indicated that there was 57% more probable to find bivalves containing *V. parahaemolyticus* in 2008 than in 2006. However in 2009 and 2010, the risk decreased significantly and the probability to find total *V. parahaemolyticus* was 2.09 and 2.26 times less than in 2006, respectively (*P* = 0.002, in both cases). Results also showed that the risk of finding *R. decussatus* containing *V. parahaemolyticus* was higher than for other species of bivalves. The probability of finding total *V. parahaemolyticus* in *R. decussatus* was 3.39 times higher than in *C. gigas* (*P* < 0.001). On the other hand, results showed that shell length of mollusks (*P* = 0.063; data not showed), temperature (*P* = 0.102), and salinity (*P* = 0.691) had no significant effect in this logistic regression model.

**Table 4:** Description of the logistic regression model selected to explain the presence/absence (P/A) of total (presence of *tlh* gene) *V. parahaemolyticus* including location, species of bivalve, length, year of campaign, temperature, and salinity.

Variables included in the selected model	*B*	OR (e^B^)	*P*-value
Fangar vs. Alfacs	0.793	2.211	<0.001
*R. decussatus* vs. *C. gigas*	1.221	3.391	<0.001
2008 vs. 2006	0.449	1.567	0.017
2009 vs. 2006	-0.738	0.478	0.002
2010 vs. 2006	-0.817	0.442	0.002
Constant	-2.159	0.115	<0.001

*Method: Forward Stepwise Conditional.*

*Model summary: -2 Log likelihood = 1116.814; Nagalkerke R Square = 0.090; Omnibus tests of model coefficients, *P* < 0.001.*

A second significant model which did not include sampling bay to explain P/A of total *V. parahaemolyticus* in bivalves was generated (*P* < 0.001; see **Table 5** for details). This model established that the presence of *V. parahaemolyticus* depends on species of mollusks, year of harvest, temperature, and salinity. Results showed that the risk to find *V. parahaemolyticus in R. decussatus* was higher than for other species of bivalves, as it was showed in the first model (above); thus the probability of finding total *V. parahaemolyticus* in *R. decussatus* was 3.02 times higher than in *C. gigas* (*P* < 0.001). Similar trends (as in the first model, **Table 4**) in total *V. parahaemolyticus* prevalence respect to the year of sampling was demonstrated using year 2006 as a reference. Results obtained in 2008 respect to 2006 indicated that the risk to find total *V. parahaemolyticus* in bivalves was 2.19 times higher in 2008 than 2006 (*P* < 0.001). In 2009, the risk was not significant respect to 2006. However in 2010, the risk decreased significantly and the probability to find total *V. parahaemolyticus* was 2.03 times less than in 2006 (*P* = 0.009).

**Table 5:** Description of the logistic regression model selected to explain the presence/absence (P/A) of total (presence of *tlh* gene) *V. parahaemolyticus* removing location.

Variables included in the selected model	*B*	OR (e^B^)	*P*-value
*R. decussatus* vs. *C. gigas*	1.106	3.023	<0.001
2008 vs. 2006	0.782	2.185	<0.001
2010 vs. 2006	-0.707	0.493	0.009
Temperature (°C)	-0.241	0.786	0.001
Salininy (ppm)	0.238	1.269	0.005
Constant	-4.085	0.017	0.232

*Method: Forward Stepwise Conditional.*

*Model summary: -2 Log likelihood = 1117.862; Nagalkerke R Square = 0.088; Omnibus tests of model coefficients, *P* < 0.001.*

Presence of total *V. parahaemolyticus* was significantly associated with water temperature (*P* = 0.001) and salinity (*P* = 0.005). The risk of finding bivalves containing *V. parahaemolyticus* decreased 1.27 times with each unit (°C) of increased temperature. Moreover, presence of total *V. parahaemolyticus* was directly correlated with salinity, where the risk to find bivalves containing *V. parahaemolyticus* increased 1.27 times with each unit (ppt) of salinity increased. Shell length (*P* = 0.135) had no significant effect in this logistic regression model.

### Multivariate Analysis of Influence of Different Factors on the Presence of Pathogenic *V. parahaemolyticus*

In all cases, no significant model was generated when the whole population of bivalves sampled was studied. However, a significant model to explain the P/A of pathogenic *V. parahaemolyticus* in bivalves contaminated with *V. parahaemolyticus* (*tlh+*) was generated (*P* < 0.001), and it showed that isolation of pathogenic *V. parahaemolyticus* (*tdh* and/or *trh* gene) is significantly associated with water salinity (*P* = 0.028). And, the risk of finding pathogenic *V. parahaemolyticus* decreased 1.45 times with every salinity unit (ppt) increase. When a logistic regression model was performed to explain the P/A of pathogenic *V. parahaemolyticus* (*tdh* gene), no effect of variables was found. However, when pathogenic *V. parahaemolyticus* (*trh* gene) was studied (*P* = 0.035), the risk of finding pathogenic *V. parahaemolyticus* (*trh+*) in mollusks contaminated with *V. parahaemolyticus* decreased 1.64 times with every ppt salinity increase (**Table 6**). Other factors, as bivalve species, length, year, and water temperature were not significantly associated with the presence of potentially pathogenic *V. parahaemolyticus*.

**Table 6:** Description of the logistic regression model selected to explain the presence/absence (P/A) of pathogenic (presence of *trh* gene) *V. parahaemolyticus* including location, species of bivalve, length, year of campaign, temperature, and salinity.

Variables included in the selected model	*B*	OR (e^B^)	*P*-value
Salinity	-0.494	0.610	0.035
Constant	15,470	5230408	0.064

*Method: Forward Stepwise Conditional.*

*Model summary: -2 Log likelihood = 122.733; Nagalkerke R Square = 0.044; Omnibus tests of model coefficients, *P* = 0.040.*

## Discussion

The present study examined intraseasonal relationships between selected environmental parameters (temperature and salinity) and the prevalences of total and pathogenic *V. parahaemolyticus* in four different species of bivalves (*C. gigas, M. galloprovincialis, R. decussatus*, and *R. philippinarum*) cultured in the Ebro delta in four different years.

In this work temperature and salinity conditions of the bays during each campaign did not suffer big fluctuations since all campaigns took place during the summer (July and August), when *V. parahaemolyticus* is more frequently present and in higher numbers. Nevertheless, differences were found between the two bays. Examination of data indicated that Alfacs Bay presented higher temperatures than Fangar Bay (1 or 2°C higher), due to the basin volume in Alfacs is about ten times larger than in Fangar, needing more time to renew its water (Camp and Delgado, 1987; Montilla et al., 1994). Alfacs bay also presented lower salinities than Fangar bay (around 1 or 2 ppt lower); the salinity of the water is influenced by differential evaporation rates and freshwater inputs from nearby agricultural (rice) fields (Camp and Delgado, 1987; Camp, 1994).

Several studies indicate that *V. parahaemolyticus* in mollusks are significantly correlated with seawater temperature; where, reported temperature ranges varied from: 10 to 33°C (DePaola et al., 2003); 9.9 to 32.7°C (Phillips et al., 2007); 14.4 to 29.2°C (Sobrinho et al., 2010); 7.7 to 29.7°C (Haley et al., 2014); and 7.9 to 25.5°C (Cruz et al., 2015).

Water temperature in Alfacs ranged between 26.93 and 27.67 and in Fangar from 25.41 to 26.37°C. In our model, the temperature was not significantly associated with total *V. parahaemolyticus* presence (**Table 4**), when the model included location. These results agree with those reported by Deepanjali et al. (2005), who observed no statistically significant correlation with tropical seawater temperature from 34 to 24°C, and, with Zimmerman et al. (2007) who did not find any correlation with temperature ranging from 22.4 to 33.8°C either. However, temperature was significantly associated (negatively) with total *V. parahaemolyticus* presence in our model (**Table 5**) when location was removed from the model, which indicated the risk of finding bivalves containing *V. parahaemolyticus* decreased 1.27 times with each unit (°C) that temperature increased. These results should be interpreted with caution because all samples were collected only during the summer season, since previous work had shown no detection of pathogenic *V. parahaemolyticus* during the other seasons of the year (data not shown), therefore it could be suggested that temperature influences *V. parahaemolyticus* levels. However, our results show that salinity in these two semi-enclosed estuarine bays is more important than summer temperature. Salinities at both sampling sites varied between 35.17 and 37.04 ppt during the sampling seasons (ranged between 35.17 and 36.01 and from 35.74 to 37.04°C in Alfacs and Fangar, respectively), which is well above the reported optimum salinity of 23 ppt for *V. parahaemolyticus* growth (Anonymous, 2005). The correlation between water salinity and total *V. parahaemolyticus* densities in bivalves suggests that salinity *per se* is an important factor for growth of this bacterium. Therefore, this logistic regression model showed (**Table 5**, removing location) the risk of finding total *V. parahaemolyticus* increases 1.27 times with each unit (ppt) of salinity increased. And these results were corroborated in 2008 (*P* = 0.001) and 2010 (*P* = 0.090). These results on a first observation are in agreement with those obtained by Zimmerman et al. (2007), who found that when salinity increased (10 to 28 and 4 to 13 ppt), densities of total *V. parahaemolyticus* increased. On the other hand, other authors as Deepanjali et al. (2005), Martinez-Urtaza et al. (2008), Sobrinho et al. (2010), Kirs et al. (2011), Cruz et al. (2015), and Young et al. (2015), did not find correlation between these parameters; whereas, others authors found the inverse correlation (DePaola et al., 2000, 2003).

As we see above, literature reports contradictory conclusions on the association between salinity and *Vibrio* spp. This apparent contradiction could be due to a narrow range and/or optimal salinity for *V. parahaemolyticus*, so any deviation (higher or lower) from 23 ppt, has an impact in its viability. Moreover, Haley et al. (2014) suggested that salinity played a role in *V. parahaemolyticus* presence, even though it did not show up as significant in their model, and/or that an unmonitored parameter present in the estuarine environments could have influenced the *V. parahaemolyticus* presence.

In our study prevalences of total *V. parahaemolyticus* in Fangar Bay (with lower temperatures and higher salinities than Alfacs Bay) were higher (16.7%) than in Alfacs Bay (11.6%; *P* = 0.005). These results are agreement with our models (**Tables 4** and **5**) which showed the risk of finding total *V. parahaemolyticus* in mollusks from Fangar Bay was 2.21 times higher than from Alfacs Bay and the environmental conditions correspond to a narrow range of high temperatures and salinities, play an important role.

Several studies have shown that *V. parahaemolyticus* levels appear to fluctuate independently from temperature and salinity, clearly showing that these factors are not the only ones that influence the bacterium’s abundance and distribution. Factors such as plankton composition, dissolved oxygen, particulate organic matter availability, and chlorophyll a, presence of fish and shellfish, as well as to levels of freshwater flows and the depth of the harvesting area though may be involved in *V. parahaemolyticus* prevalence (McCarter, 1999; Phillips et al., 2007; Zimmerman et al., 2007, Vezzulli et al., 2009; Deter et al., 2010; Julie et al., 2010; Ceccarelli et al., 2013).

Previous studies which indicated that only 1–3% of the environmental strains produce TDH or contain the *tdh* gene (Kelly and Stroh, 1988; DePaola et al., 1990; Nair et al., 2007; Cruz et al., 2015) or they are not detected (Shirai et al., 1990; Honda and Iida, 1993). In the present study pathogenic *V. parahaemolyticus* prevalences observed and analyzed by years show that higher prevalence was registered in Fangar Bay (8.6%, of which 6.7% were *tdh*-positive) in 2008, and this result may be due to an increased prevalence of total *V. parahaemolyticus* that year. No significant differences were observed in Alfacs (*P* = 0.747), in contrast to Fangar Bay (*P* = 0.004); And, although this results may be due to a type II error, failing to find differences where they exist, it is unlikely taking into account the considerable amount of data taking analyzed in this study.

Our finding are in agreement with those of DePaola et al. (2003) who indicated a higher prevalence (12.8%) of *tdh*-positive *V. parahaemolyticus* in Alabama oysters determined by direct plating, and also with those of Deepanjali et al. (2005) who found similar prevalences of *tdh*-positive *V. parahaemolyticus* (10.2%) in Oysters from India by colony hybridization. In the present study the highest prevalence observed in *V. parahaemolyticus* strains harboring the *trh* gene was 1.4% in both bays. Other studies did not detect the presence of *V. parahaemolyticus* carrying the *trh* virulence gene in their samples (Kirs et al., 2011; Cruz et al., 2015). While, Deepanjali et al. (2005) have indicated a high prevalence of *trh-*positive *V. parahaemolyticus* in oysters.

When logistic regression model to explain the P/A of each gene (*tdh/trh*) was performed in relation with the different factors analyzed, only *trh*-positive *V. parahaemolyticus* correlates (negatively) with water salinity (**Table 6**), suggesting that strains carrying this gene are more sensitive to salinity or that the optimal salinity for strains containing the *trh* gene is lower than for other strains of this species.

## Conclusion

Our results indicate temporal and spatial variations in the prevalences of total and pathogenic *V. parahaemolyticus* in both bays and in the bivalve mollusks. Apparently, *V. parahaemolyticus* populations in bivalves are controlled quantitatively and qualitatively by different factors. It seems unlikely that selective filtration of non-pathogenic to pathogenic *V. parahaemolyticus* could affect for the magnitude of different concentrations in bivalves at the two sites (Genthner et al., 1999; Anonymous, 2005). Higher prevalences of *V. parahaemolyticus* were registered in *R. decussatus* and in *R. philippinarum*, which may indicate either a potential host effect or an effect due to culture systems, since oysters and mussels are grown in suspended ropes, clams are grown on the floor bed. It is also possible that the filtration rate of clams, under the sampling conditions, were closer to optimal when compared to mussels and oysters which would contribute to higher filtration rate in clams with consequent accumulation of bacteria.

Present study collected a considerable amount of data on the presence of total and pathogenic *V. parahaemolyticus* over a long period of time which makes this data robust. Although these data were collected in one area of the Mediterranean coast, similar environmental conditions of those of the Mediterranean coast can be found in places like California (USA), Southern Australia, Central Chile, and the western cape of South Africa. Both Australia and the USA have legislation for the safety of bivalves for consumption which includes pathogenic vibrios indicating that present data can be very useful when performing a risk analysis for assessing the consequences of *V. parahaemolyticus* presence in commercial bivalves in the Mediterranean countries.

## Conflict of Interest Statement

The authors declare that the research was conducted in the absence of any commercial or financial relationships that could be construed as a potential conflict of interest.
